# *Sp*Crus2 Glycine-Rich Region Contributes Largely to the Antiviral Activity of the Whole-Protein Molecule by Interacting with VP26, a WSSV Structural Protein

**DOI:** 10.3390/md19100544

**Published:** 2021-09-27

**Authors:** Yue Wang, Chao Zhang, Wen-Hong Fang, Hong-Yu Ma, Xin-Cang Li

**Affiliations:** 1 Guangdong Provincial Key Laboratory of Marine Biotechnology, Shantou University, Shantou 515063, China; wangyue19930913@163.com; 2 Key Laboratory of East China Sea Fishery Resources Exploitation, Ministry of Agriculture, East China Sea Fisheries Research Institute, Chinese Academy of Fishery Sciences, Shanghai 200090, China; fwenhong@163.com; 3 Institute of Tropical Aquaculture and Fisheries, Universiti Malaysia Terengganu, Kuala Nerus 21030, Malaysia; 4 Chongqing Three Gorges Vocational College, Wanzhou, Chongqing 404155, China; shandongneau@aliyun.com

**Keywords:** *Scylla paramamosain*, crustin, glycine-rich region (GRR), cysteine-rich region (CRR), WSSV, binding and antiviral activities

## Abstract

Crustins are cysteine-rich cationic antimicrobial peptides with diverse biological functions including antimicrobial and proteinase inhibitory activities in crustaceans. Although a few crustins reportedly respond to white spot syndrome virus (WSSV) infection, the detailed antiviral mechanisms of crustins remain largely unknown. Our previous research has shown that *Sp*Crus2, from mud crab *Scylla paramamosain*, is a type II crustin containing a glycine-rich region (GRR) and a cysteine-rich region (CRR). In the present study, we found that *SpCrus2* was upregulated in gills after WSSV challenge. Knockdown of *SpCrus2* by injecting double-stranded RNA (*dsSpCrus2*) resulted in remarkably increased virus copies in mud crabs after infection with WSSV. These results suggested that *SpCrus2* played a critical role in the antiviral immunity of mud crab. A GST pull-down assay showed that recombinant *Sp*Crus2 interacted specifically with WSSV structural protein VP26, and this result was further confirmed by a co-immunoprecipitation assay with *Drosophila* S2 cells. As the signature sequence of type II crustin, *Sp*Crus2 GRR is a glycine-rich cationic polypeptide with amphipathic properties. Our study demonstrated that the GRR and CRR of *Sp*Crus2 exhibited binding activities to VP26, with the former displaying more potent binding ability than the latter. Interestingly, pre-incubating WSSV particles with recombinant *Sp*Crus2 (r*Sp*Crus2), rGRR, or rCRR inhibited virus proliferation in vivo; moreover, r*Sp*Crus2 and rGRR possessed similar antiviral abilities, which were much stronger than those of rCRR. These findings indicated that *Sp*Crus2 GRR contributed largely to the antiviral ability of *Sp*Crus2, and that the stronger antiviral ability of GRR might result from its stronger binding activity to the viral structural protein. Overall, this study provided new insights into the antiviral mechanism of *Sp*Crus2 and the development of new antiviral drugs.

## 1. Introduction

Antimicrobial peptides (AMPs) are evolutionarily conserved immune-defense components found in all living organisms, ranging from bacteria to mammals [1,2]. AMPs are multifunctional molecules exhibiting broad-spectrum activity against bacteria, fungi, protozoa, and viruses. The notable characteristic of AMPs is that they are small, amphipathic proteins with hydrophobic and cationic properties [2,3]. The special amphipathic structure of AMPs enables the killing of target organisms by disrupting their cell membrane. The antibacterial mechanism of AMPs relatively differs from that of antibiotics, which easily lead to the development of drug-resistant bacteria through long-term abuse. Therefore, AMPs are considered ideal therapeutic reagents of the future.

As the major effectors in the innate immune system of crustaceans, numerous AMPs have been identified. At least four AMP families, namely, crustins, anti-lipopolysaccharide factors (ALFs), penaeidins, and lysozymes, have been well-characterized [4,5,6,7]. Crustins are cationic, cysteine-rich, antibacterial peptides first identified in crustaceans with similar structural characteristics as follows: a signal peptide (SP), a variable region at the N-terminus, and a whey acidic protein (WAP) domain with eight cysteine residues forming a four-disulfide core at the C-terminus [5]. Smith et al. proposed the first classification scheme for crustins, and three types of crustins have been grouped based on their distinct sequence and structural characteristics [5]. Apart from an SP, type I crustins contain a cysteine-rich domain and a WAP domain, type II crustins have an extra glycine-rich region (GRR) at the *N*-terminus compared with type I crustins, and type III crustins comprise a Pro-Arg-rich region and a WAP domain. Later, double WAP domain-containing molecules and novel insect crustins have been proposed as types IV and V crustins, respectively [8].

Currently, viral infection remains one of the most serious disease threats to the cultivation of crustaceans [9]. White spot syndrome virus (WSSV) is a highly destructive pathogen that severely restricts the healthy development of crustacean-farming industries worldwide [10]. WSSV has wide-ranging host species and belongs to the new genus *Whispovirus* of the *Nimaviridae* family. It is an enveloped virus with a 305-kb double-stranded circular DNA genome, and numerous WSSV structural proteins have been identified by proteomic methods [11]. Among them, VP15 is considered as a core protein of WSSV involved in packing the viral genome [12]. VP19 is a major envelope protein and is required for the envelope coating of this virus [13]. Meanwhile, VP26 is a special envelope protein (tegument protein) acting as a matrix-like linker protein between the viral envelope and nucleocapsid, which is regarded as a key factor in the envelopment of the WSSV virion [14]. All these three structural proteins participate in the assembly of WSSV.

AMPs in crustaceans display broad-spectrum antimicrobial activity. Previous studies have shown that type I crustins are cysteine-rich peptides comprising a cysteine-rich domain and a WAP domain. They exhibit antibacterial activity only against Gram-positive bacteria [5,15]. Compared with type I crustins, type II crustins have an extra GRR and have antibacterial activities against Gram-positive and Gram-negative bacteria [5]. The GRR itself exhibits antimicrobial activity and extends the antibacterial spectrum of type II crustins [16,17]. Some types I and II crustins have been found to respond to WSSV infection participating in antiviral immunity [18,19,20]. However, the antiviral mechanisms of these crustin molecules are largely unknown. Moreover, as the signature sequence of type II crustins, whether some GRRs interact with WSSV particles and participate in antiviral immune responses remains unclear.

*Sp*Crus2 is a type II crustin identified from the mud crab *Scylla paramamosain*. Our previous research has demonstrated that *Sp*Crus2 displays broad antimicrobial activity and that its GRR enhances its antibacterial activity [17]. In the present study, we found it was upregulated after WSSV challenge, suggesting that it may participate in the antiviral immune response. To further reveal the likely antiviral mechanisms of *Sp*Crus2, we first analyzed the sequence characteristics of *Sp*Crus2 GRR and those from other type II crustins. We then investigated the binding activity of its functional regions (GRR and cysteine-rich region (CRR)) to major structural proteins of WSSV and finally evaluated its antiviral activities. Through these assays, we attempted to elucidate the correlation of the antiviral activities of *Sp*Crus2 and its functional regions with its typical sequence characteristics or with its binding activities to viral proteins. This study provided new insights into the antiviral mechanism of *Sp*Crus2, as well as a reference sequence and theoretical basis for the development of new antiviral drugs.

## 2. Results

### 2.1. Bioinformatic Analysis of SpCrus2 GRR

**Figure 1:**
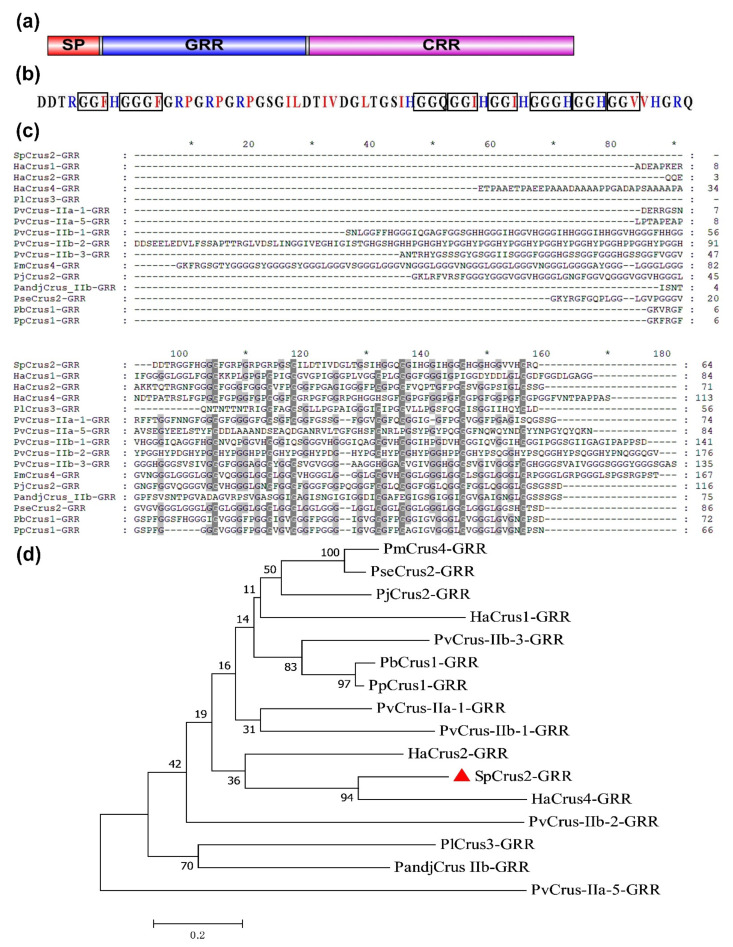
Bioinformatic analysis of *SpCrus2*. (**a**) Schematic structure of *Sp*Crus2. SP, signal peptide; GRR, glycine-rich region; CRR, cysteine-rich region. (**b**) *Sp*Crus2 GRR sequence. The common short glycine-rich polypeptide repetitions were boxed. Positively charged amino acids (H and R) are indicated in blue color, and hydrophobic amino acids (F, P, I, L and V) are indicated in red color. (**c**) Multiple alignment of *Sp*Crus2 GRR with GRRs of retrieved representative crustins. (**d**) Phylogenetic analysis of *Sp*Crus2 GRR and other crustin GRRs by MEGA 7.0. Bootstrap analysis (1000 replications) was conducted and the node values were automatically produced and shown in this tree. *Sp*Crus2 GRR is marked with a red triangle.

Our previous studies have demonstrated that *Sp*Crus2 protein comprises three regions [17]: an SP (1–17 AA), a GRR (18–81 AA), and a CRR (82–164 AA) (Figure 1a). Here, we further analyzed the major sequence characteristics (the number of AA, MW, pI, and glycine content) of *Sp*Crus2 GRR, as well as the GRRs of other representative crustins in crustaceans. Table 1 shows that the number of AAs of GRRs ranged from 21 to 176, the MWs ranged from 1.99 to 17.9 kDa, the pIs ranged from 3.56 to 12.48, and the glycine contents ranged from 15.5% to 67.9%. Among them, the *Sp*Crus2 GRR was a cationic polypeptide with a pI of 9.44, and it had 12 positively charged AAs (R and H) and 15 hydrophobic AAs (F, P, I, L, and V) (Figure 1b). These AAs defined *Sp*Crus2 GRR as an amphipathic polypeptide with hydrophobic and cationic properties. We also noticed that the glycine content of *Sp*Crus2 GRR was about 40%, and that most glycine residues were in the form of short glycine-rich repeats (tri- and tetra-), such as GGF, GGH, GGG, GGGF, and GGGH, which are the common polypeptide repetitions in other GRRs.

Similarity analysis showed that *Sp*Crus2 GRR had the highest similarity with *Ha*Crus4 GRR, but the identity was only 26.4%, and a low identity of 23.33% existed among representative crustin GRRs (Figure 1c). To analyze the likely evolutionary relationships among them, a phylogenetic tree was constructed using *Sp*Crus2 GRR and other GRRs of crustacean crustins. In this tree, no meaningful large clusters were observed because their node values were very low (<60) (Figure 1d), suggesting that the amino acid sequences of GRRs were diverse. However, *Sp*Crus2-GRR and *Ha*Crus4-GRR formed a meaningful branch with a node value of 94, indicating that they may possess certain special biological functions different from those of other GRRs.

### 2.2. SpCrus2 Was Upregulated after WSSV Challenge 

**Figure 2:**
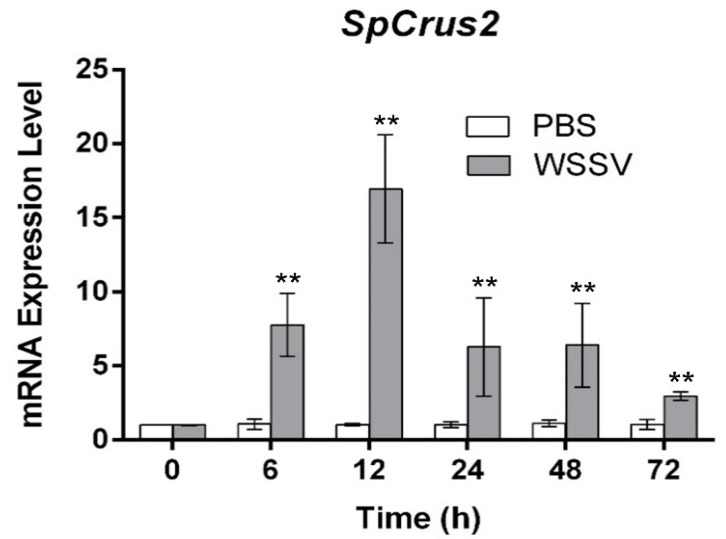
Expression pattern of *SpCrus2* in gills after WSSV challenge. The WSSV particles or PBS (negative control) were injected into crabs. Error bars represented ± standard deviation of three independent assays. Asterisks indicated significant differences from the control (**, *P* < 0.01).

Our previous study has revealed that *SpCrus2* is highly expressed in gills [17]. Herein, the time-course expression profile of *SpCrus2* in gills after WSSV challenge was further analyzed. We found that *SpCrus2* was significantly upregulated from 6 to 72 h post-WSSV challenge, and it reached the maximum level at 12 h post-challenge (Figure 2). This result suggested that *SpCrus2* may participate in the antiviral immunity of mud crab.

### 2.3. SpCrus2 Knockdown Facilitated WSSV Proliferation

**Figure 3:**
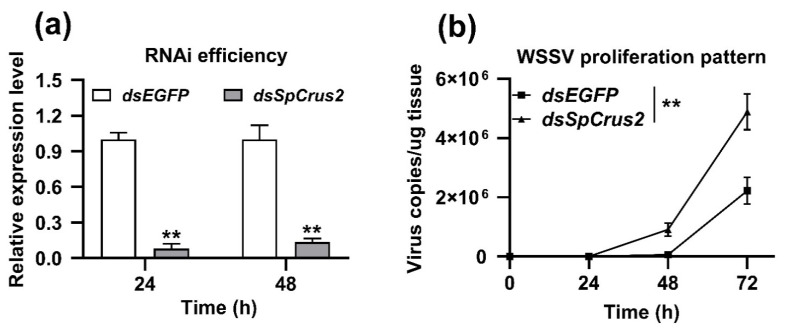
Silencing *SpCrus2* enhanced WSSV replication in gills. (**a**) RNAi efficiency of *SpCrus2* in gills was evaluated by qRT-PCR at 24 and 48 h after injection with dsRNA (*dsEGFP* or *dsSpCrus2*). *dsEGFP* was injected as negative control. Asterisks indicated extremely significant differences (**, *P* < 0.01) compared with the control. (**b**) WSSV was inoculated at 24 h after dsRNA injection. The number of viral copies in gills was assessed through qPCR at different time points (0, 24, 48, and 72 h) after WSSV injection. The virus copies showed the extremely significant differences (**, *P* < 0.01) at 48 and 72 h post-infection with WSSV.

To further verify the antiviral role of *SpCrus2* in the process of WSSV infection, mud crabs were initially injected with *dsSpCrus2* to silence *SpCrus2* and then challenged with WSSV. As shown in Figure 3a, *SpCrus2* was successfully knocked down by injecting *dsSpCrus2*, because the expression level of this gene at 24 and 48 h after injection with *dsSpCrus2* was less than 25% of that in the control crabs treated with *dsEGFP*. Subsequently, the proliferation profiles of WSSV in dsRNA-treated crabs were investigated by analyzing viral-genome copies (DNA virus) through qPCR. Results showed that the quantities of WSSV in the gills of *SpCrus2*-silenced crabs were much higher than those in control crabs at 48 or 72 h post-viral infection (Figure 3b). Collectively, these data clearly suggested that *SpCrus2* was a critical antiviral factor that could significantly inhibit WSSV proliferation in vivo.

### 2.4. WSSV Structure Proteins Were Recombinantly Expressed in a Soluble Form

**Figure 4:**
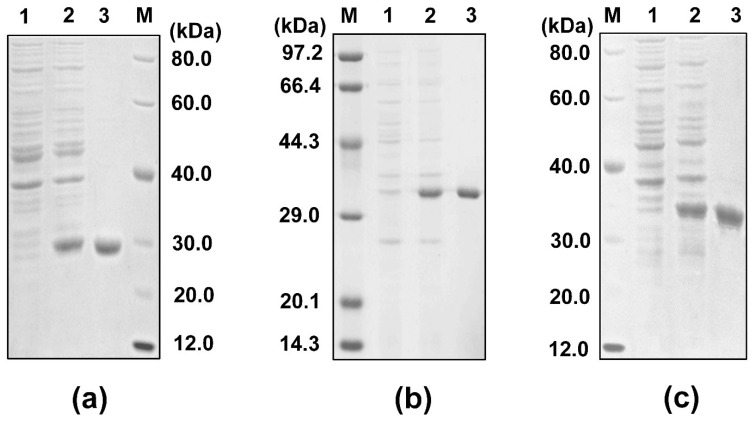
SDS-PAGE analysis of viral structural proteins expressed in *E. coli*. Lane M, protein marker; Lane 1, total proteins of *E. coli* cells containing expression vector without IPTG induction; Lane 2, total proteins of *E. coli* cells containing expression vector with IPTG induction; Lane 3, purified rVP15 (**a**), rVP19 (**b**), or rVP26 (**c**).

To investigate the possible antiviral mechanism of *Sp*Crus2, three major structural proteins of WSSV, VP15, VP19, and VP26 were recombinantly expressed in *Escherichia coli*. Each recombinant protein comprised a mature peptide and a His tag-containing vector peptide encoded by pET32a (for VP15 and VP19) or by pET30a (for VP26). After the viral protein-containing bacterial cells were lysed and centrifuged, all viral proteins were detected in the supernatants, indicating that they were expressed in a soluble form. Accordingly, the proteins were purified further with Ni-NTA His-binding resin through affinity chromatography. The predicted MWs of the recombinant VP15 (rVP15), rVP19 and rVP26 were 28.2, 29.3, and 24.9 kDa, respectively, which approximately agreed with the sizes of the major bands that appeared in the corresponding purified protein lanes (Figure 4). 

### 2.5. SpCrus2 Interacted Specifically with the WSSV Structural Protein VP26

**Figure 5:**
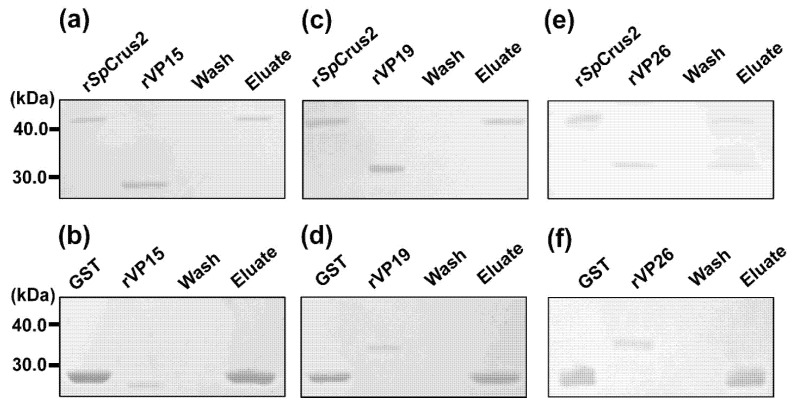
A GST Pull-down assay was conducted to examine the binding activity of *Sp*Crus2 with viral structural proteins. r*Sp*Crus2 and viral structural protein (rVP15, rVP19, or rVP26) were mixed, and then incubated with Glutathione Sepharose 4B resin. The gels were visualized after staining with Coomassie blue. r*Sp*Crus2 interacted with rVP26 (**e**) but not with rVP15 (**a**) or rVP19 (**c**). GST served as the control in this assay (**b**,**d**,**f**).

**Figure 6:**
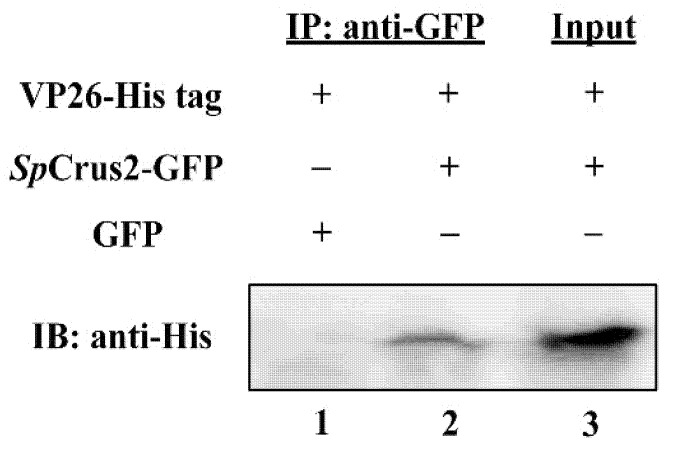
*Sp*Crus2 specifically interacted with WSSV structural protein VP26 in S2 cells. S2 cells were co-transfected with plasmids pAc5.1B-VP26-His and pAc5.1B-*Sp*Crus2-GFP (or pAc5.1B-GFP). Lanes 1 and 2: experimental samples, cell lysates were immunoprecipitated (IP) with anti-GFP affinity resins; the IP complexes were subjected to Western blotting with anti-His antibody. Lane 3: input, cell lysates were subjected to Western blotting with anti-His antibody serving as positive control.

A GST pull-down assay was performed to test the binding ability of *Sp*Crus2 to viral proteins (VP15, VP19, and VP26). Results showed that r*Sp*Crus2 could specifically bind to rVP26, but it did not have any apparent binding ability to rVP15 and rVP19 (Figure 5). The binding activity of *Sp*Crus2 to VP26 was further validated by a co-immunoprecipitation (Co-IP) assay. Results showed that *Sp*Crus2 could specifically bind to GFP-fused VP26 but not to GFP protein (Figure 6). These findings clearly demonstrated that *Sp*Crus2 specifically interacted with VP26, suggesting that VP26 may be a potential target component that could be recognized by *Sp*Crus2.

### 2.6. SpCrus2 GRR Showed Stronger Binding Affinity to VP26 Than Its CRR

**Figure 7:**
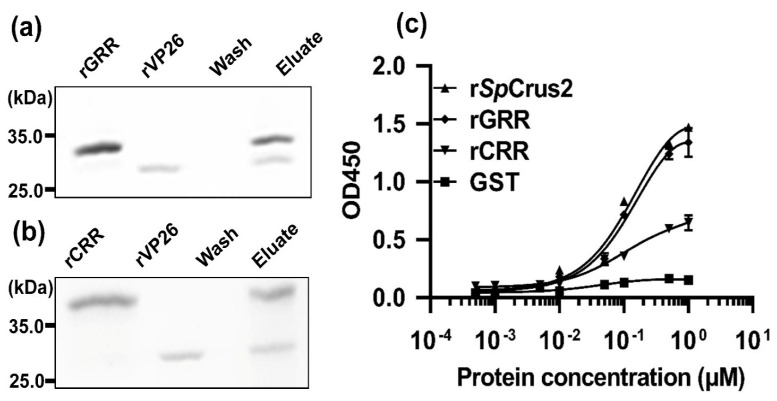
Both the GRR and CRR of *Sp*Crus2 bound to VP26 in varying degrees. A GST pull-down assay was conducted to test the binding activity of *Sp*Crus2 GRR or CRR to VP26. The results were visualized by staining with Coomassie blue. Either the rGRR (**a**) or rCRR (**b**) interacted with rVP26. (**c**) ELISA was conducted to evaluate the binding affinity of *Sp*Crus2, GRR, or CRR with VP26. Plates were coated with rVP26. r*Sp*Crus2, rGRR, or rCRR was serially diluted and then added onto wells of the coated plates. These three recombinant proteins exhibited remarkable binding activities to rVP26, and r*Sp*Crus2 and rGRR displayed stronger binding activity to rVP26 than rCRR.

In the present study, the mature peptide of *Sp*Crus2 was divided into two functional regions, the GRR and CRR, based on its sequence and structural characteristics. Given that *Sp*Crus2 specifically interacted with viral protein VP26, these two functional regions may play distinct roles in the binding activity to VP26. Thus, rGRR and rCRR, together with rVP26, were applied to a GST pull-down assay to determine which functional region was responsible for the specific interaction between *Sp*Crus2 and VP26. Results demonstrated that either rGRR or rCRR could specifically bind to rVP26 (Figure 7a,b). Furthermore, enzyme-linked immunosorbent assay (ELISA) was performed to evaluate the degree of their individual binding ability to VP26. As shown in Figure 7c, we observed that r*Sp*Crus2 and its two regions all displayed binding activities to rVP26 in a concentration-dependent manner. Interestingly, r*Sp*Crus2 and rGRR had similar binding activities, and their binding activities to rVP26 were much stronger than that of rCRR. These results revealed that *Sp*Crus2 GRR instead of the CRR played a dominant role in the interaction between *Sp*Crus2 and VP26.

### 2.7. SpCrus2 GRR Exhibited Stronger Inhibitory Activity on WSSV Proliferation Than Its CRR

**Figure 8:**
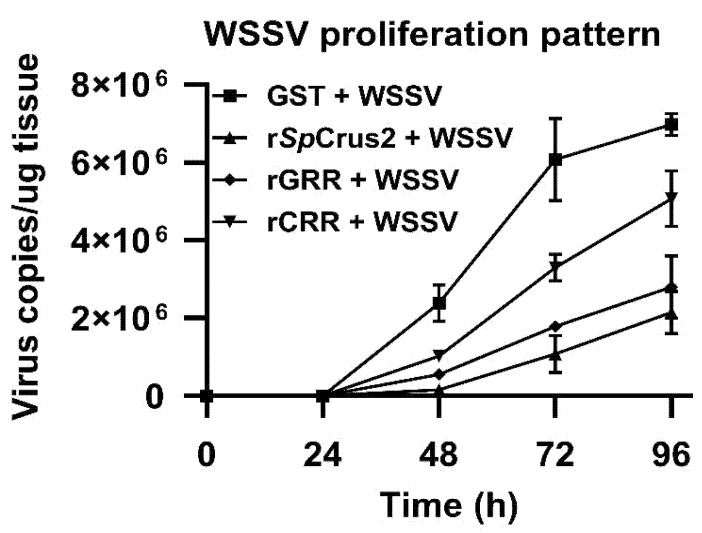
*Sp*Crus2 and its two functional regions significantly inhibited WSSV proliferation. Prepared WSSV particles were pre-incubated with r*Sp*Crus2, rGRR, rCRR, or with GST tag protein and then injected into crabs. The number of virus copies in gills at each time point was determined by qPCR.

Considering that *Sp*Crus2 GRR and CRR could interact with VP26 exhibiting different binding activities, we speculated that these two proteins may suppress WSSV proliferation to varying degrees. To clarify the potential inhibitory activity exerted by *Sp*Crus2 on viral replication, the proliferation profiles of WSSV in gills after the pre-incubation of viral particles with rGRR or rCRR were analyzed. Compared with WSSV particles pre-incubated with GST protein, the virus pre-incubated with either rGRR or rCRR significantly inhibited WSSV proliferation at 48, 72, and 96 h post-injection (Figure 8). Moreover, rGRR displayed a stronger inhibitory activity against WSSV than rCRR because the pre-incubation of virus with rGRR resulted in much lower virus copies at 48, 72, and 96 h post-infection. The inhibitory activity of *Sp*Crus2 against WSSV was subsequently investigated. Figure 8 shows that coating WSSV particles with r*Sp*Crus2 dramatically inhibited virus replication in vivo, and r*Sp*Crus2 and rGRR shared a similar inhibitory activity. This result suggested that *Sp*Crus2 GRR played a dominant role in the antiviral activity of the whole protein, whereas CRR exerted only a synergistic effect on this process.

## 3. Discussion

Crustins are a class of cationic AMPs with diverse biological functions, including antibacterial, protease-inhibitory, and antiviral activities [21]. However, the detailed antiviral molecular mechanisms of crustins are unclear. In the present study, we found that *Sp*Crus2 GRR was a cationic polypeptide with a highly flexible molecular structure, and that it interacted specifically with the viral structural protein VP26. Besides, the knockdown of *SpCrus2* significantly facilitated WSSV replication, and pre-incubating WSSV particles with *Sp*Crus2 GRR dramatically suppressed virus proliferation. Moreover, *Sp*Crus2 GRR showed much stronger inhibitory activity against WSSV proliferation than its CRR. These findings revealed that *Sp*Crus2 GRR contributed largely to the antiviral ability of *Sp*Crus2 in mud crab.

In crustaceans, a single species can express a series of crustins with diverse antimicrobial activities against different pathogens. For instance, six crustins have been characterized in *S. paramamosain*, and they displayed broad antimicrobial activities [17,22,23,24,25]. Based on the sequence and structural differences, these six crustins have been divided into two classes (types I and II). Compared with type I crustins, type II ones have an additional GRR, which is the signature motif of type II crustins and confers increased biological activities to this type of molecule [16,17]. Moreover, GRR could reportedly be considered as a core characteristic for crustin classification [26]. Accordingly, in the present study, we determined the sequences of most type II crustins in crustaceans by database searching and further analyzed the sequence characteristics of their GRRs. We found that the glycine contents of GRRs dramatically varied from 15.5% to 67.9%, and most of glycine residues were present in the form of short glycine-rich repeats. Considering that the numbers and types of short glycine-rich repeats relatively differed in each GRR, different compositions of various short glycine-rich repeats resulted in a more complicated or diverse sequence structure. The sequence diversity of GRRs implied that these glycine-rich polypeptides may display diverse biological functions. *Sp*Crus2 GRR shared a low similarity with most crustacean GRRs and contained at least five different short glycine-rich repeats. This finding suggested that it may have certain biological activity, making *Sp*Crus2 a special molecule with distinctive physiological function.

Glycine-rich proteins with a high glycine content have been reported in many different creatures. The GRRs of these proteins provide discrete secondary structures, which are very flexible and can promote themselves and adjacent domains to interact with their target molecules [27,28]. A similar conclusion has been drawn by a study on a shrimp type II crustin, crustin*Pm*1, whose GRR-deleted mutant has a much lower antibacterial activity than itself [16]. Our previous research has also revealed that the GRRs of crustins enhance the antibacterial activities of whole proteins and broaden their antimicrobial spectrum [17]. Although the GRR and CRR of *Sp*Crus2 have antibacterial activities, the antibacterial ability of the latter is much stronger than that of the former. In the present study, we further demonstrated that these two functional regions of *Sp*Crus2 exhibited antiviral activities, and interestingly, *Sp*Crus2 GRR had a stronger antiviral activity than its CRR. These findings indicated that *Sp*Crus2 GRR was a multifunctional glycine-rich polypeptide with antibacterial and antiviral activities. The diverse antimicrobial activities possessed by *Sp*Crus2 GRR may be due to its broad binding activities toward various microbes because most microbes are negatively charged on the surface. These negative charges enable easy interaction with a cationic polypeptide, such as *Sp*Crus2 GRR. However, this phenomenon does not mean that *Sp*Crus2 GRR can bind to each kind of microbe because the interaction of the GRR’s positively charged residues with the anionic components of target microbes are specific, and the space distances between the GRR and microbial components differ, thereby affecting the affinity between GRR and microbes. Anyway, *Sp*Crus2 GRR is a glycine-rich cationic polypeptide with flexible structure that can interact specifically with several different components of microbes, which may be the intrinsic cause leading to the multiple biological functions of *Sp*Crus2 GRR, as well as the whole protein.

Some crustins are reportedly involved in antiviral immune responses [18,19,20]. In *S. paramamosain*, previous studies have shown that *Sp*Crus2 and *Sp*Crus6 are significantly upregulated after WSSV challenge; however, *Sp*Crus3, *Sp*Crus4, and *Sp*Crus5 do not exhibit any expression change post-viral infection [17,23,24,25]. *Sp*Crus6 also inhibits the proliferation of WSSV by interacting with the viral structural protein VP26 [25]. Given that *Sp*Crus6 is a type I crustin and the mature peptide has only a CRR (including a cysteine-rich domain and a WAP domain) without GRR, the antiviral activity of *Sp*Crus6 should be exerted by its CRR. In the present study, we further demonstrated that *Sp*Crus2 CRR interacted with VP26, displaying antiviral activity. The findings revealed that some CRRs of crustins played a role in anti-WSSV immune responses by interacting with the specific viral component. In fact, we notice that the cysteine-distribution patterns in the CRRs of *Sp*Crus2 and *Sp*Crus6 differ even though these two CRRs contain the same number of cysteine residues [17,25]. Thus, the structures of these two CRRs and their binding manner to WSSV may differ despite the fact that they both interact with VP26. Considering that only a few type I crustins have been shown to possess antiviral activity, the exact antiviral mechanism of type I crustins (or CRRs) remains to be elucidated.

AMPs are a group of small proteins first defined based on their antibacterial activities. The following studies revealed that they are more than antibacterial proteins; some of them are multifunctional molecules with additional antiviral and/or antifungal activities [19,29]. In crustaceans, at least four different AMP families including ALFs, crustins, lysozymes, and penaeidins are well-studied [4,5,6,7]. A few members of ALF and LYZ families have been shown to restrict WSSV proliferation by interacting with viral-structure proteins or by disrupting virus integrity. For instance, *Cq*ALF disrupts the integrity of the WSSV envelope, leading to dramatically reduced WSSV infectivity in crayfish *Cherax quadricarinatus* [30]. *Lv*LYZ1 exhibits anti-WSSV activity in shrimp by binding to several viral-structure proteins [9]. Although a few crustins reportedly respond to WSSV infection [18,19,20], the antiviral molecular mechanisms exerted by crustins, especially type II crustins, remain largely unclear. In the present study, we found that *Sp*Crus2 GRR had a stronger antiviral activity than CRR. It also had a stronger binding activity to VP26 than CRR. By contrast, our previous study has shown that *Sp*Crus2 GRR has a lower affinity to bacterial components and a weaker antimicrobial activity than its CRR [17]. Therefore, the antiviral and antimicrobial activities of these two polypeptides were significantly affected by their respective binding activities to different microbial components. Moreover, the stronger antiviral ability of *Sp*Crus2 GRR may result from its more potent binding activity to VP26. With regard to the possible molecular mechanism leading to the potent binding and antiviral abilities of *Sp*Crus2 GRR, we conjectured that its flexible structure and unique amphipathic property played a critical role in its biological processes. The flexible structure of *Sp*Crus2 GRR can facilitate the interaction of this amphipathic polypeptide with the special envelope component VP26. Though VP26 is a cationic protein, we found the AAs on the outer surface (AAs from Asp68 to Ile201) of its trimer form an anionic polypeptide with the pI of 5.03. The negatively-charged surface of VP26 provides potential binding sites for the positively charged GRR. Moreover, the binding of the GRR to viral protein can induce its neighbor region CRR and VP26 to make easier contact and interact with each other. Thus, we believe that *Sp*Crus2 GRR plays a dominant role in the antiviral process mediated by *Sp*Crus2.

In the present study, we found that *Sp*Crus2 GRR was a glycine-rich amphipathic polypeptide with multiple short glycine-rich repeats. This special structure gave this polypeptide a stronger binding affinity with the structural protein VP26, which contributed largely to the potent antiviral ability of *Sp*Crus2. *Sp*Crus2 CRR also had lower binding activity to VP26, leading to a lower antiviral activity, which may be the intrinsic cause of why some type I crustins had antiviral functions considering that they shared a similar structure to *Sp*Crus2 CRR. This study provided new insights into understanding the underlying antiviral mechanisms of *Sp*Crus2 and a theoretical basis for the development of new antiviral drugs.

## 4. Materials and Methods

### 4.1. Reagents and Chemicals

RNAiso Plus, First-Strand cDNA Synthesis Kit, and SYBR Premix Ex Taq Polymerase were purchased from TaKaRa Biotech (Dalian, China). TIANamp Marine Animals DNA Kit was obtained from Tiangen Biotech (Beijing, China). GST Fusion Protein Purification Kit and His-tagged Protein Purification Kit were purchased from GenScript (Nanjing, China).

### 4.2. Mud Crab Challenge and Tissue Collection

Mud crabs (approximately 150 g each) purchased from a farm in Chongming County (Shanghai, China) were cultured in aerated seawater in tanks (400 L) for 7 days prior to the experiments. Healthy mud crabs were randomly selected and divided into two groups. A total of 1 × 10^5^ WSSV particles in 100 µL of PBS were injected into the base of the fifth leg of mud crabs, and an equal volume of PBS served as a control. After immune stimulation at 0, 6, 12, 24, 48, and 72 h, mud crabs were anesthetized by soaking them in ice for approximately 10 min, and their gills were collected by dissecting and rinsing with sterile PBS to isolate total RNA and study the *SpCrus2* expression pattern. For each group, at least three crabs were sampled to eliminate individual differences. Another two batches of previously isolated RNA samples were used to eliminate the differences among batches.

### 4.3. Total RNA Isolation and cDNA Synthesis

RNAiso Plus reagent was used to extract the total RNA from gills. DNase I (Promega Co., Madison, WI, USA) was used to remove the contaminated genomic DNA during total RNA extraction. First-Strand cDNA Synthesis Kit was used to synthesize cDNA templates with the total RNAs according to the manufacturer’s instructions.

### 4.4. Bioinformatic Analysis

ProtParam (https://web.expasy.org/protparam/, accessed on 30 April 2020) was used to predict and analyze the physical and chemical properties of *Sp*Crus2 GRR, including relative molecular weight, theoretical isoelectric point, and amino acid composition. The similarities of *Sp*Crus2 GRR to other crustin GRRs were analyzed using ClustalX 2.0 program (http://www.ebi.ac.uk/tools/clustalw2, accessed on 3 May 2020). Multiple sequence alignment of proteins was performed using Bioedit software (http://www.mbio.ncsu.edu/bioedit/bioedit.html, accessed on 4 May 2020). The phylogenetic tree was constructed using MEGA 7.0 software [31].

### 4.5. Quantitative Real-Time Polymerase Chain Reaction (qRT-PCR)

qRT-PCR was conducted to determine the expression profile of *SpCrus2* after WSSV challenge in a real-time thermal cycler Quantstudio 6 Flex (Applied Biosystems, Foster City, CA, USA) following a previous protocol [17]. A pair of primers (*Sp*Crus2RF and *Sp*Crus2RR; Table 2) was designed to produce a 121 bp amplicon of *SpCrus2*. Another pair of primers (18SRF and 18SRR; Table 2) was used to amplify a 121 bp fragment of 18S rRNA as a reference. The total volume was 20 µL (10 µL of 2 × SYBR Premix Ex Taq, 2 µL of cDNA, and 4 µL of each primer). qRT-PCR was programmed as follows: 95 °C for 3 min, 40 cycles at 95 °C for 10 s and 60 °C for 60 s. and a melt from 60 °C to 95 °C. All tests were performed thrice with individual templates and then analyzed with the algorithm of 2^−^^△△CT^ [32]. Unpaired *t*-test was used to determine significant differences (**, *P* < 0.01; *, *P* < 0.05).

### 4.6. RNA Interference (RNAi) of SpCrus2

RNAi assay was conducted using a previously described method with slight modifications [33]. In a typical procedure, a partial *SpCrus2* DNA fragment was amplified by PCR with primers linked with the T7 promoter (*Sp*Crus2iF and *Sp*Crus2iR; Table 2) and used as the template to produce double-stranded RNA (dsRNA) with an in vitro T7 transcription kit (Takara, Dalian, China). *EGFP dsRNA* (*dsEGFP*) was generated as the control in the same way using the primers listed in Table 2 (EGFPiF and EGFPiR). Mud crabs (approximately 20 g each) were randomly divided into two groups. Each crab was administered with an intraperitoneal injection at the base of the fifth leg with *dsSpCrus2* (40 μg) or with the same amount of *dsEGFP*. At 24 and 48 h post-injection, gills were collected for RNA extraction, and RNAi efficiency was then determined by qRT-PCR with the total RNA. Experiments were performed independently three times. For each test, at least five crabs per group were used. Unpaired Student’s *t*-test was used to determine significant differences (**, *P* < 0.01; *, *P* < 0.05).

### 4.7. Recombinant Expression and Purification of Viral Proteins, SpCrus2, GRR, and CRR

Three pairs of gene-specific primers (VP15EF and VP15ER, VP19EF and VP19ER, and VP26EF and VP26ER; Table 2) were designed to amplify the corresponding DNA sequences of WSSV structure proteins (VP15, VP19, and VP26). Each amplified DNA fragment was completely digested using restriction enzymes (*Eco*R I/*Xho* I for VP15 or VP19, *Bam*H I/*Xho* I for VP26) and then inserted into a pET32a vector (for VP15 or VP19) or into a pET30a vector (for VP26). The recombinant plasmid (pET32a-VP15, pET32a-VP19, or pET30a-VP26) was transformed into *E*. *coli* Rosetta (DE3) competent cells. Protein expression was induced by adding isopropyl-β-d-thiogalactoside to a final concentration of 0.5 mM at 37 °C for 6 h. The bacteria were then pelleted through centrifugation and resuspended in PBS containing 0.1% Triton X-100 for probe sonication lysis. Each recombinant protein was fused with a His tag and purified with Ni-NTA His-Binding Resin. Protein concentration was determined by the Bradford method. The GST-tagged *Sp*Crus2, GRR, and CRR, as well as GST protein, were overexpressed and purified with glutathione-Sepharose 4B resin by using the bacterial strains developed in our previous study [17].

### 4.8. Profiles of WSSV Proliferation Affected by RNAi of SpCrus2 or by Protein Pre-Incubation

Mud crabs (~20 g) were randomly divided into two groups (six crabs per group), and each crab was injected with dsRNA (*dsSpCrus2* or *dsEGFP*) to knockdown the expression of *SpCrus2*. After validating that *SpCrus2* was silenced, each crab was injected with the same amount of purified WSSV virions (2 × 10^4^ copies). The *dsEGFP*-treated mud crabs served as the control. At 0, 24, 48, and 72 h post-WSSV injection, gills were sampled to extract genomic DNA for WSSV quantification. The WSSV copies in gills at each time point were calculated according to a previously developed method [25]. The profiles of WSSV proliferation affected by pre-incubating *Sp*Crus2 and its functional regions were also investigated. Mud crabs (~100 g each) were randomly divided into four groups (six crabs per group). Prepared WSSV virions (1 × 10^5^ copies) were pre-incubated with r*Sp*Crus2, rGRR, rCRR, or with GST protein (200 μg/mL) at room temperature for 30 min and then injected into each crab in the corresponding groups. For each group, the gills from at least three mud crabs were sampled at 0, 24, 48, 72, and 96 h post-injection to calculate the virus copies by qPCR through the same method.

### 4.9. GST Pull-Down Assay

The GST pull-down assay was performed according to a previously reported method with slight modifications [25]. A total of 150 μL of glutathione–Sepharose 4B resin (50% beads slurry) after washing with PBS was incubated with a mixture of GST-fused *Sp*Crus2 (15 μg) and each recombinant viral protein (rVP15, rVP19, or rVP26; 15 μg) for 2 h at room temperature. GST served as the control. After incubation, the samples were washed thoroughly with PBS, and then the proteins were eluted by adding PBS containing 10 mM reduced glutathione. The binding activity of rGRR and rCRR with these three viral proteins was further analyzed with the same method. The elution samples were subjected to 12.5% SDS-PAGE and analyzed after staining the gel with Coomassie blue.

### 4.10. Plasmid Constructions, Cell Culture, and Co-IP Assay

Two pairs of gene-specific primers (Table 2) were designed to obtain the individual-coding mature-peptide sequences of *Sp*Crus2 and VP26. The harvested fragment of *Sp*Crus2 was cloned into a pAc5.1B-GFP vector to generate the plasmid pAc5.1B-*Sp*Crus2-GFP. The DNA fragment of VP26 was cloned into a pAc5.1/V5-His B vector to generate pAc5.1B-VP26-His. *Drosophila* Schneider 2 (S2) cells (Catalog no. R690-07) were cultured at 28 °C in Schneider’s *Drosophila* medium (Gibco) supplemented with 10% fetal bovine serum (Invitrogen, Carlsbad, CA, USA). In the Co-IP assay, cells were seeded onto six-well plates (Corning, VA, USA), and transfections were performed 24 h later. Cells were co-transfected with 2 μg of pAc5.1B-*Sp*Crus2-GFP plasmid (or pAc5.1B-GFP) and 2 μg of pAc5.1B-VP26-His per well by using FuGENE HD Transfection Reagent (Promega) according to the user manual. pAc5.1B-GFP served as a negative-control plasmid. At 48 h post-transfection, the cells were lysed with NP40 lysate (Beyotime) and then centrifuged at 12,000 rpm for 20 min at 4 °C. A small fraction of the supernatants (20 μL) was sampled as input, and the remaining supernatants were incubated with GFP-trap Agarose beads (KT-HEALTH, Changzhou, China) overnight at 4 °C with rotation. The beads were collected by centrifugation, washed three times with PBS, and then resuspended in 1× SDS sample buffer. After boiling for 10 min, the resultant samples were separated by SDS-PAGE and analyzed by Western blot using mouse anti-His tag monoclonal antibody (GenScript).

### 4.11. ELISA

ELISA was used to investigate the binding abilities of *Sp*Crus2 and its two functional regions (GRR and CRR) with VP26 following a previous method with slight modifications [34]. In a typical procedure, a total of 100 µL (200 µg/mL) rVP26 was used to coat each well of a flat-bottom microtiter plate (high-binding microtiter plate; Greiner) at 37 °C for 2 h. The plates were then blocked with 200 μL of BSA (2 mg/mL) for 2 h at 37 °C, followed by washing four times with 0.05% Tween-20 in TBS (TBST). After washing, serially diluted r*Sp*Crus2, rGRR, rCRR, or GST (0.0005–1 μM in TBS containing 0.1 mg/mL BSA) was added to the microplates. The plates were incubated at 37 °C for 3 h before washing the wells with TBST four times. Subsequently, peroxidase-conjugated mouse monoclonal anti-GST antibody (1:5000 dilution in TBS with 1 mg/mL BSA) was added, and the plates were incubated for 2 h under the same conditions. After rinsing the wells with TBST four times, the color reaction was developed with 0.01% 3,3′,5,5′-tetramethylbenzidine (Sigma) liquid substrate in citric acid-Na_2_HPO_4_ buffer. The reaction was stopped with 2 M H_2_SO_4_, and absorbance was recorded at 450 nm wavelength by using a microtiter plate reader (Tecan, Switzerland). GST served as a negative control. All assays were performed in triplicate.

## Figures and Tables

**Table 1:** Basic sequence information of GRRs of crustins retrieved in crustaceans.

Species	Gene Names	AAs Numbers	MWs (kDa)	pIs	Gly Contents	NCBI Accession Number of Genes
*Scylla paramamosain*	*Sp*Crus2	64	6.1	9.44	40.6	AUV47162
*Hyalella azteca*	*Ha*Crus1	84	7.3	4.19	47.6	XP_018011791
	*Ha*Crus2	71	6.3	9.99	45.1	XP_018011805
	*Ha*Crus3	26	2.3	3.8	30.8	XP_018011808
	*Ha*Crus4	113	10.1	4.69	31.9	XP_018017888
*Pacifastacus leniusculus*	*Pl*Crus3	56	5.3	6.74	32.1	ABP88044
*Penaeus vannamei*	*Pv*Crus-IIa-1	74	6.6	9.51	50.0	MT375570
	*Pv*Crus-IIa-2	61	4.9	8.75	57.4	AAL36890
	*Pv*Crus-IIa-3	63	5.5	8.59	42.9	AFV77524
	*Pv*Crus-IIa-4	62	5.9	11.7	41.9	MT375573
	*Pv*Crus-IIa-5	84	9.1	4.1	15.5	MT375574
	*Pv*Crus-IIa-6	56	5.0	6.99	33.9	MT375575
	*Pv*Crus-IIa-7	70	6.9	7.16	35.7	MT375576
	*Pv*Crus-IIa-8	50	4.26	7.06	42.0	MT375577
	*Pv*Crus-IIa-9	35	3.0	5.98	37.1	MT375578
	*Pv*Crus-IIa-10	53	4.0	7.89	67.9	MT375579
	*Pv*Crus-IIa-11	50	4.52	5.38	40.0	MT375580
	*Pv*Crus-IIb-1	141	12.5	6.84	46.8	MT375581
	*Pv*Crus-IIb-2	176	17.9	6.33	31.2	MT375582
	*Pv*Crus-IIb-3	135	10.8	8.51	54.8	MT375583
	*Pv*Crus1	49	4.1	10.84	55.1	AAL36892
	*Pv*Crus2	61	5.0	8.75	55.7	AAL36894
	*Pv*Crus3	67	5.4	8.75	58.2	AAL36895
*Penaeus monodon*	*Pm*Crus1	42	3.9	12.48	38.1	ACQ66004
	*Pm*Crus2	62	5.6	12.0	46.8	XP_037772780
	*Pm*Crus4	167	13.0	10.53	63.5	ACQ66005
	*Pm*Crus5	63	6.1	6.25	38.1	ACP40176
	*Pm*Crus6	33	3.1	12.3	33.3	ABW82154
	*Pm*Crus7	52	4.7	5.38	40.4	ACL97375
	*Pm*Crus8	57	5.1	5.38	40.4	ABV25094
*Penaeus japonicus*	*Pj*Crus1	88	7.5	9.99	56.8	BAD15062
	*Pj*Crus2	116	9.8	9.99	57.8	BAD15063
	*Pj*Crus3	108	9.2	9.99	57.4	BAD15064
	*Pj*Crus4	104	8.8	9.99	57.7	BAD15065
	*Pj*Crus5	80	6.8	9.99	56.2	BAD15066
	*Pj*Crus9	49	4.5	12.48	51.0	BBC42585
*Pandalus japonicus*	*Pandj*Crus IIb	75	6.4	4.03	36.0	AGU01544
	*Pandj*Crus IIc	21	2.1	12	23.8	AGU01545
*Panulirus japonicus*	*PanujCrus1*	30	2.8	6.26	36.7	ACU25382
	*PanujCrus2*	38	3.6	6.92	36.8	ACU25383
	*PanujCrus3*	32	3.3	5.74	31.2	ACU25384
*Penaeus setiferus*	*Pse*Crus1	22	1.99	9.75	27.3	AAL36896
	*Pse*Crus2	86	6.7	8.6	62.8	AAL36897
*Penaeus subtilis*	*Psu*Crus1	44	3.7	8.75	54.5	ABO93323
*Penaeus brasiliensis*	*Pb*Crus1	72	6.0	8.75	55.6	ABQ96197
*Penaeus paulensis*	*Pp*Crus1	66	5.4	11	56.1	ABM63361
*Penaeus chinensis*	*Pc*Crus2	28	2.76	12	25.0	ACZ43782
*Penaeus schmitti*	*Psc*Crus1	64	5.2	8.6	57.8	ABM63362
*Penaeus indicus*	*Pi*Crus1	33	3.1	4.65	33.3	ACV84092
*Paralithodes camtschaticus*	*Pcam*Crus1	26	2.6	3.56	19.2	ACJ06765

**Table 2:** Sequences of the primers used in this study.

Primers	Sequence (5′–3′)
**Real-time PCR**	
*Sp*Crus2RF	TAGACGGACTTACTGGGAGCAT
*Sp*Crus2RR	GGGACATTCACACCGCACT
**18s rRNA**	
18SRF	CAGACAAATCGCTCCACCAAC
18SRR	GACTCAACACGGGGAACCTCA
**RNAi**	
*Sp*Crus2iF	GCGTAATACGACTCACTATAGGGGATGACACCAGGGGGGGCT
*Sp*Crus2iR	GCGTAATACGACTCACTATAGGGTTAGGCGGAAGGTTTACAC
EGFPiF	GCGTAATACGACTCACTATAGGGTGGTCCCAATTCTCGTGGAC
EGFPiR	GCGTAATACGACTCACTATAGGGCTTGAAGTTGACCTTGATGCC
**Protein expression**	
VP15EF	TACTCA*GAATTC*ATGACAAAATACCCCGAGAT
VP15ER	TACTCA*CTCGAG*TTAACGCCTTGACTTGCGGAC
VP19EF	TACTCA*GAATTC*ATGGCCACCACGACTAACAC
VP19ER	TACTCA*CTCGAG*ATCCCTGGTCCTGTTCTTAT
VP26EF	TACTCA*GGATCC*ACACGTGTTGGAAGAAGCGT
VP26ER	TACTCA*CTCGAG*CTTCTTCTTGATTTCGTCCT
**Co-immunoprecipitation (Co-IP)**	
*Sp*Crus2CF	CG*GAATTC*GCCATGGACACCAGGGGGGGCTTCCAC
*Sp*Crus2CR	CCG*CTCGAG*CGGGCGGAAGGTTTACACACGGGATGG
VP26CF	GG*GGTACC*GCCACCATGGGTGTTGGAAGAAGCGTC
VP26CR	GC*TCTAGA*CTCTTCTTCTTGATTTCGTCCTTG

Underlined nucleotides indicate the locations of restricted endonucleases.
